# ADP-ribosylation signalling and human disease

**DOI:** 10.1098/rsob.190041

**Published:** 2019-04-17

**Authors:** Luca Palazzo, Petra Mikolčević, Andreja Mikoč, Ivan Ahel

**Affiliations:** 1 Institute of Protein Biochemistry, National Research Council, Via Pietro Castellino 111, 80131 Naples, Italy; 2 Division of Molecular Biology, Ruđer Bošković Institute, Bijenička cesta 54, 10000 Zagreb, Croatia; 3 Sir William Dunn School of Pathology, University of Oxford, South Parks Road, OX1 3RE Oxford, UK

**Keywords:** ADP-ribosylation, signalling, translational medicine

## Abstract

ADP-ribosylation (ADPr) is a reversible post-translational modification of proteins, which controls major cellular and biological processes, including DNA damage repair, cell proliferation and differentiation, metabolism, stress and immune responses. In order to maintain the cellular homeostasis, diverse ADP-ribosyl transferases and hydrolases are involved in the fine-tuning of ADPr systems. The control of ADPr network is vital, and dysregulation of enzymes involved in the regulation of ADPr signalling has been linked to a number of inherited and acquired human diseases, such as several neurological disorders and in cancer. Conversely, the therapeutic manipulation of ADPr has been shown to ameliorate several disorders in both human and animal models. These include cardiovascular, inflammatory, autoimmune and neurological disorders. Herein, we summarize the recent findings in the field of ADPr, which support the impact of this modification in human pathophysiology and highlight the curative potential of targeting ADPr for translational and molecular medicine.

## 1. Introduction

Uni- and multicellular organisms rely on multiple dynamic molecular processes dictating cellular growth, cell division, prompt adaptation to environmental changes and survival. A functional and dynamic communication between cellular macromolecules is essential to control these fundamental biological processes. At the molecular level, proteins and small molecules are responsible for orchestrating these cellular responses. Cells have evolved mechanisms able to regulate dynamically proteins' functions through chemical modifications. In this regard, post-translational modifications (PTMs) can efficiently and very rapidly control a multitude of cellular processes in a time-dependent fashion by affecting the conformation, activity, stability, interactions and the sequestration of proteins to cellular compartments and organelles [1].

So far, more than 300 PTMs have been described; each one is involved in a range of fundamental cellular and biological processes. Functional alterations in the proteins governing PTM systems are frequently dysregulated in human disease [1].

Among the PTMs, there is ADP-ribosylation (ADPr). ADPr is the transfer of a single or multiple ADP-ribose unit(s) from nicotinamide adenine dinucleotide (NAD^+^) onto target protein substrates. Importantly, ADP-ribose nucleotide units can be also transferred onto nucleic acids and small molecules, such as on acetyl chemical groups to produce *O*-acetyl-ADP-ribose (OADPR) during de-acetylation reactions [2–4].

Although ADPr of proteins was first described in the early 1960s, our understanding of the cellular processes regulated by ADPr is still in its infancy [5–8]. Indeed, strikingly little is known about most of the proteins involved in ADPr and the governed signalling pathways. Such a gap in the knowledge also translates into a lack of understanding of many potentially related pathogenic mechanisms. Yet the therapeutic modulation of ADPr is emerging as a strategy with high potential in the clinic of certain human cancer types [9,10].

However, an in-depth understanding of molecular networks controlled by ADPr can not only further potentiate current clinical strategies, but also impact on the treatment of many other human diseases with no available therapy identified so far. Herein we discuss the most recent discoveries available in the scientific community supporting the central role of ADPr in the pathophysiology of many acquired and hereditary human diseases (summarized in table 1) and highlight the outcomes of the pharmacological modulation of ADPr for the clinical treatment of these disorders.

**Table 1.** Alterations of ADPr genes associated with human inherited pathologies.

gene	gene alteration	disease/disorder	references
transferases			
PARP9	overexpression	B-aggressive lymphoma	[11,12]
		breast cancer	[13]
PARP14	overexpression	B-aggressive lymphoma	[11,14]
		sarcoma	[15]
		asthma	[16]
		hepatocellular carcinoma	[17]
PARP15	overexpression	B-aggressive lymphoma	[11]
readers/erasers			
ALC1 (CHD1L)	overexpression	hepatocellular carcinoma	[18]
		breast cancer	[19]
		colorectal carcinoma	[20]
ARH1	missense mutations	lung, breast and colon cancers	[21]
ARH3	truncations/mutations	neurodegenerative diseases	[22,23]
GDAP2 (MacroD3)	point mutations	ataxia, progressive spasticity and dementia	[24]
MacroD1	overexpression	endometrial carcinoma	[25]
		gastric carcinoma	[26]
		colorectal carcinoma	[27,28]
		breast carcinoma	[29,30]
MacroD2	single-nucleotide polymorphisms	autism	[31–33]
	microdeletion Int 5	kabuki syndrome	[34,35]
	locus deletions	various cancers	[36,37]
	deletions, missense mutations	colorectal cancer	[38]
TARG1	premature stop codon	neurodegeneration	[39]

## 2. ADP-ribosyl transferases

ADPr is carried out by transferase enzymes that, based on the homology of their catalytic domain with bacterial toxins, are classified in two enzyme superfamilies: the cholera toxin-like ADP-ribosyl transferases (ARTCs) and the diphtheria toxin-like ADP-ribosyl transferases (ARTDs) [2,40,41]. These two classes of enzymes share an evolutionarily conserved protein fold, called ADP-ribosyl transferase (ART) domain [40,41]. The ART protein fold is characterized by two central β-sheets, one anti-parallel sheet containing three to five β strands, and one sheet composed of four to five β strands [40–42].

**Figure 1.**
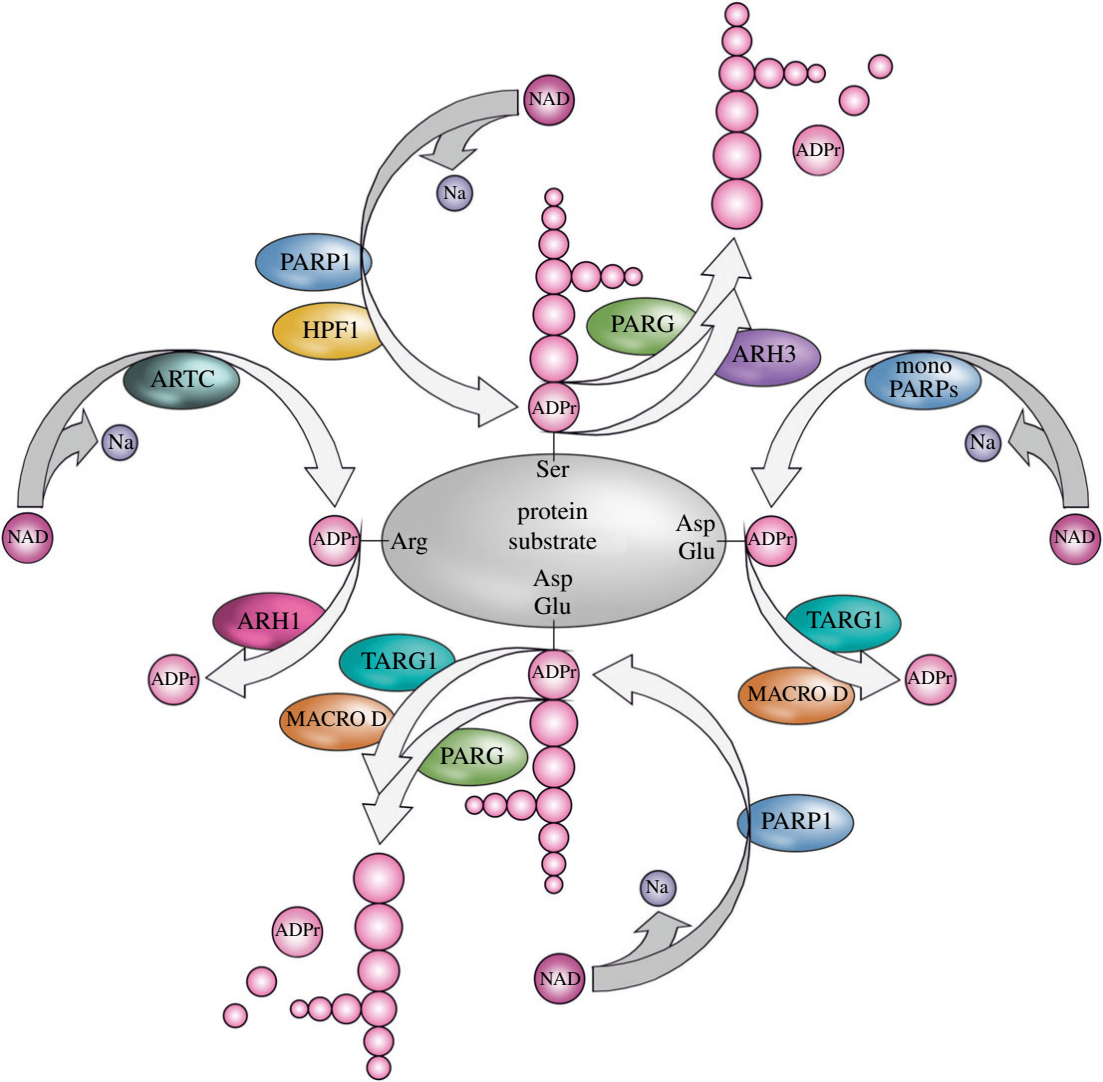
Enzymes and mechanisms of protein ADP-ribosylation. NAD, nicotinamide adenine dinucleotide; Na, nicotinamide; ADPr, ADP-ribose.

Three crucial amino acids within the ART domain define the affiliation to cholera or diphtheria toxin-like superfamilies, the R-S-E and H-Y-E triads, respectively. The first two amino acids in the triad are important for the NAD^+^ binding, while the common glutamate functions in catalysis [40–42]. ARTCs and ARTDs also differ for their specificity to target distinct amino acids. Most of the characterized ARTCs target protein substrates on arginine residues in proteins through an *N*-glycosidic bond producing arginine-ADPr (Arg-ADPr; figure 1). The founding member of ARTC family is the cholera toxin from *Vibrio cholerae*. Cholera toxin modifies arginine 187 of the stimulatory Gs*α* subunit of heterotrimeric G protein. ADPr of Gs*α* leads to constitutive activation of cyclic AMP-signalling pathway and, in turn, a dramatic efflux of ions and water from infected enterocytes, leading to watery diarrhoea [43,44].

ARTD group of transferases most commonly modify acidic groups [45] (figure 1). The founding member of ARTD family is the diphtheria toxin, an exotoxin secreted by *Corynebacterium diphtheriae*, which catalyses the modification of the eukaryotic elongation factor-2 (EF-2) at a modified amino acid called diphthamide, thus inhibiting the translation machinery of the host [44,46]. For further details about bacterial ADP-ribosyl transferase toxins (bARTTs), refer to §9.

Four members of ARTC superfamily are expressed in humans (ARTC1, ARTC3, ARTC4, and ARTC5) and six in mice (Artc1, Artc2.1, Artc2.2, Artc3, Artc4 and Artc5). ARTC1, ARTC2, ARTC3 and ARTC4 enzymes are bound to the cellular plasma membrane by a glycosyl-phosphatidyl-inositol (GPI) anchor, while ARTC5 is an extracellular secreted enzyme. ARTC1, ARTC2 and ARTC5 show mono-ADP-ribosyl transferase activity and modify arginine side chains of protein substrates (figure 1). On the contrary, ARTC3 and ARTC4 lack the R-S-E motif in the active centre and therefore are probably inactive enzymes [47,48].

Mainly, extracellular or plasma membrane-residing proteins are substrates of Arg-ADPr, such as P2X7 and haemopexin [47,48]. However, several studies revealed Arg-ADPr of intracellular proteins as well (e.g. BIP, GAPDH and tubulin) [49–52], though the ARTs responsible for intracellular Arg-ADPr remain largely unknown.

Seventeen members of the ARTD superfamily have been identified in mammals and are known as poly(ADP-ribose) polymerases (PARPs) [3,41,53]. PARPs most commonly transfer ADP-ribose onto aspartic/glutamic acids (Asp/Glu-ADPr), through ester linkages, and on serine (Ser-ADPr) residues through *O*-glycosylation [54,55] (figure 1). Several PARPs can produce chains of ADP-ribose polymers (also called poly(ADP-ribose), thus abbreviated as PAR), where repeating single ADP-ribose units (up to 200 in length) are linked via unique *O*-glycosidic ribose-ribose bonds [45,56–58]. This type of modification is generally named poly(ADP-ribosyl)ation (PARylation). Well-characterized PARPs able to generate PARylation are PARP1, PARP2, Tankyrase-1 and Tankyrase-2 [45]. However, the remaining human PARP members are instead only capable of transferring a single ADP-ribose group to their target proteins, thus producing mono(ADP-ribosyl)ation (also abbreviated as MARylation) [45,59,60] (figure 1).

## 3. ADP-ribosyl hydrolases

ADPr is a fully reversible PTM. Two unrelated protein families show hydrolytic activity against proteins modified by ADPr, with diverse target specificity; the ADP-ribosyl-acceptor hydrolases (ARHs) and the macrodomain-containing enzymes.

Mg^2+^-dependent ADP-ribosyl-acceptor hydrolases (ARHs) are classified as DraG-like fold-containing proteins, based on the homology encountered with the bacterial dinitrogenase reductase-activating glycohydrolase (DraG). Bacterial DraG homologues have been described as mono(ADP-ribosyl) hydrolases that control nitrogen fixation by counteracting the arginine modifying ART activity of DraT [61]. Three ARH members are found in mammals: ARH1 (also named ADPRH), ARH2 (also named ADPRHL1) and ARH3 (also named ADPRHL2). While an enzymatic activity has not been identified for ARH2, ARH1 and ARH3 have distinct substrate specificities [62–64]. As seen for the bacterial DraG proteins, ARH1 reverses Arg-ADPr synthesized by both mammal endogenous ARTCs and bacterial toxins [64] (figure 1). Indeed, *Arh1*-deficient mice show enhanced sensitivity to cholera toxin infection [65]. By contrast, ARH3 shows high activity on *O*-glycosidic bonds and is the only known enzyme possessing hydrolytic activity against Ser-ADPr [66] (figure 1). Interestingly, ARH3 is inhibited by the metabolite ADP-ribosyl arginine, suggesting a cross talk between ADPr systems [67].

Macrodomain-containing proteins share a common ADP-ribose recognition domain, which is called macrodomain. The macrodomain is an ADP-ribose binding unit that plays crucial roles in the sensing and hydrolysis of ADPr in different cellular contexts [68]. Macrodomains are found in vertebrates as well as in many bacteria, archaea, viruses and plants, suggesting their evolutionary conservation and wide utility [69]. Depending on the type, macrodomains can exhibit the ability to bind ADP-ribose or PAR or OADPR (a metabolite released from the sirtuin-mediated NAD^+^-dependent deacetylation reaction). In addition, some macrodomains also act as ADP-ribosyl hydrolases [42,68–71]. Nevertheless, several macrodomain-containing proteins have been suggested to bind RNA intermediates instead of ADP-ribose [69,72].

Among the 12 macrodomain-containing proteins encoded by the human genome, only four exhibit catalytic activity [2,3,68,69]. The poly(ADP-ribosyl)glycohydrolase (PARG) is the only macrodomain-containing protein that efficiently cleaves PAR chains, though, it is unable to remove the terminal ADP-ribose linked to protein substrates [73]. Conversely, MacroD1 and MacroD2 of the MacroD subfamily of proteins as well as Terminal ADP-ribose glycosylhydrolase 1 (TARG1/C6orf130 or OARD1) specifically hydrolyse protein MARylation on acidic residues [39,74,75] (figure 1).

## 4. PARP1

PARP1 is the best-studied PARP enzyme, which is also the most ubiquitous and abundant PARP protein [57]. Together with PARP2 and PARP3, PARP1 belongs to the DNA-dependent nuclear PARPs group whose catalytic activity is potently stimulated by DNA breaks [10,76,77]. However, over the years, PARP1 functions have been expanded with roles in DNA damage repair as well as transcription, chromatin structure and metabolism [76,78–80]. Thus, PARP1 appears to be involved in both basal processes and response to cellular stresses with implications in human disease, particularly in cancer. For instance, PARP1 functions in DNA damage repair are the most attractive strategy to induce selective cell death in DNA damage repair-deficient cancers. Novel and specific structure-based chemicals acting as inhibitors of DNA damage PARPs (most notably PARP1) have been developed and under experimentation for treatment of pathological conditions [9,10,81,82]. The topic of PARP1 inhibitors in cancer will not be discussed in detail in this review.

The regulation of PARP1 activity is essential. A distinct PARP1 interacting protein milieu may play a crucial role in the fine-tuning of PARP1 when it functions in specific physiological processes or stress conditions. One of the best-characterized PARP1 accessory proteins is Histone PARylation Factor 1 (HPF1), which is required during the switch from basal conditions to stress response [83]. HPF1 has a central role in triggering PARP1-dependent ADPr of histone proteins as well as of many other DNA damage-related proteins following genotoxic stresses [83,84]. In the presence of DNA damage, HPF1 directs PARP1 to modify target proteins on serine residues within conserved motifs usually preceded by lysine residues (KS motifs) [54,84,85] (figure 1). Notably, most of the DNA damage-inducible ADPr is lost in the absence of HPF1. Importantly, PARP1 can still modify itself and other proteins on acidic residues in both DNA-damaged and undamaged cells in the absence of HPF1 [85].

In addition to the modification of DNA repair proteins, PARP1/HPF1-dependent Ser-ADPr targets include many other proteins involved in the maintenance of the genome stability. Indeed, in response to oxidative DNA damage, Ser-ADPr has been found linked to RNA processing, chromatin modification, splicing, transcription factors and mitotic proteins [84,86,87]. Interestingly, Ser-ADPr often overlaps with phosphorylation sites on proteins; such as Ser-ADPr of Ser10 on Histone H3, a well-known mitotic marker [54,85,88]. Proteome-wide studies have further expanded this observation, showing that Ser-ADPr occupies serine phosphorylation sites of many proteins that are also target of the mitotic regulators Aurora A and Aurora B kinases [86]. In addition, Ser-ADPr has been shown to compete with other PTMs by steric hindrance, in particular with modifications targeting the histone tails [88,89].

### 4.1. Role of PARP1 in human diseases

Although *PARP1* genetic alterations are not associated with any known inherited disease, PARP1 is involved in the pathogenesis of many human disorders. For instance, depletion of NAD^+^ induced by PARP1 over-activation as well as excessive synthesis of PAR associates with ischaemia reperfusion injury, myocardial infarction and neurodegenerative disorders [90–94]. These disorders as well as many other acute or chronic pathological processes share a common pathogenic mechanism, which involves the production of reactive oxygen (ROS) or nitrogen species (NOS) followed by DNA damage and PARP1 activation. For instance, PARP1 was found activated in myocardial sections of patients with circulatory shock, with a degree of PARP activation correlating with the degree of myocardial dysfunction. Similar observations were made in circulating leucocytes in patients affected by myocardial infarction and therapeutic revascularization [95–97]. Moreover, PARP1 activation was shown in brain specimens of patients who died of stroke or brain ischaemia attributable to cardiac arrest, as well as in patients affected by brain trauma [98,99]. Finally, there is evidence for a boost of PARylation mediated by PARP1 in autoimmune (e.g. systemic lupus erythematosus) and inflammatory diseases (e.g. colitis), as well as in human atherosclerotic plaques, microvessels and lymphocytes of type 2 diabetic patients [100–108].

This body of information suggests a central role for PARP1 in human disorders. Indeed, the chemical modulation of PARP1 can be proposed to ameliorate or treat many pathological conditions, from cardiovascular, inflammatory and autoimmune diseases to neurological disorders. We next describe the role of PARP1 activation and the effects of its inhibition in the pathogenesis of neurological disorders, such as in a rare cerebellar ataxia caused by biallelic loss of function mutations of XRCC1, Parkinson's disease (PD), amyotrophic lateral sclerosis (ALS) and Alzheimer's disease (AD) [94,109,110].

### 4.2. PARP1 in neurological disorders

The base excision repair (BER) X-ray repair cross-complementing 1 (XRCC1) protein is a molecular scaffold protein that is recruited by PAR and PARP1 on DNA damage foci. The BRCT domain of XRCC1 mediates its recruitment on DNA damage sites, and it is vital for the assembly of DNA single-strand break repair (SSBR) protein factors [111–115]. Importantly, several DNA-end processing enzymes recruited by XRCC1 are mutated in human ataxias, such as the spinocerebellar ataxia with axonal neuropathy-1 (SCAN1; mutated in TDP1), ataxia oculomotor apraxia-1 (AOA1; mutated in aprataxin) and ataxia oculomotor apraxia-4 (AOA4; mutated in PNKP) [116–121]. Furthermore, compound heterozygous mutations in human *XRCC1* gene were shown to be responsible for ocular motor apraxia, axonal neuropathy and progressive cerebellar ataxia [109]. Mechanistically, in the presence of DNA damage, XRCC1 depletion results in severe delays in DNA SSBR repair and hyper-recombination phenotypes, which are accompanied by PARP1 hyper-activation followed by elevated levels of nuclear ADPr. The hyper-recombination as well as the cerebellar ataxia phenotype in *Xrcc1* knockout mice is rescued by *Parp1* gene deletion but not by enzymatic inhibition of PARP1. Thus, preventing the binding of PARP1 to DNA but not its enzymatic inhibition can be exploited for the therapeutic treatment of clinical cerebellar ataxias associated with unrepaired SSBs [109].

The genetic or enzymatic modulation of PARP1 has been also proposed for other common neurodegenerative diseases, such as PD, ALS and AD. These neurological disorders have a common pathogenic mechanism, which is characterized by aggregation of cytotoxic proteins, elevated levels of oxidative stress followed by DNA damage, PARP1 activation and excess of cellular levels of PAR.

In PD, intracellular monomeric α-synuclein assembles into higher-ordered protein aggregates that can spread from cell to cell [122]. Aggregates of α-synuclein activate nitric oxide synthase followed by production of NOS, which in turn cause DNA damage and activation of PARP1, and nuclear production of PAR. In a pathogenic loop, PAR is transported into the cytosol where it binds α-synuclein and further accelerates fibrillization and misfolding of the cytotoxic protein. Accumulation of pathologic α-synuclein ultimately leads to cell death via parthanatos and neuronal dysfunction. Inhibition of PARP activity or *Parp1* gene deletion fully mitigates neuron-to-neuron transmission of pathologic α-synuclein and neurotoxicity; thus, PARP inhibitors (PARPi) can be exploited as therapeutic intervention for PD [110].

The liaison between protein aggregations, ROS formation, DNA damage and PARP1 activation has been also largely shown in AD. A peptide of 39–42 amino acids (Aβ) is the major component of protein aggregates present in AD senile plaques. Aβ is produced by the sequential proteolytic processing of the amyloid precursor protein (APP) by β- and γ-secretases [123]. Genetic and/or environmental factors are responsible for an imbalance between production and clearance of A β, which in turn leads to Aβ oligomerization and production of higher-order soluble assemblies and protofibrils and fibrils [123]. Through the impairment of the mitochondrial electron transport and the interaction with metal ions (Cu^2+^, Zn^2+^ and Fe^2+^), the aggregation of Aβ leads to ROS production and PARP1 activation [124–131]. The chemical inhibition of PARP1 blocks the accumulation of PAR and the morphological transformation in microglia-induced Aβ [130].

In ALS, the normally nuclear RNA/DNA-binding protein TDP-43 redistributes in the cytoplasm of affected neurons and glial cells, and forms phosphorylated protein aggregates [132,133]. TDP-43 and other proteins mutated in ALS (e.g. Ataxin-2) are a component of stress granules (SGs). SGs are cytoplasmic membraneless structures composed of RNAs and associated proteins structures, which form well cellular-defined zones of stalled translation complexes in response to a variety of environmental stresses that interfere with mRNA translation [134,135]. Among the cellular stresses inducing SGs there are heat shock, glucose deprivation, oxidative stress and viral infection [136,137]. Importantly, several PARPs and PAR itself have been shown to localize and regulate SGs formation; for instance, PARP1, Tankyrase-1 (also known as PARP5a), PARP12, the two PARP13 splice variants (PARP13.1 and PARP13.2) and PARP15 [138–142]. In ALS, motor neurons in the spinal cord show high levels of nuclear staining of PAR, suggesting massive PARP1 activation. In turn, PARP1 activity facilitates the nuclear export and the cytoplasmic aggregation of TDP-43 by an unknown mechanism. Indeed, the specific inhibition of PARP1 by Veliparib mitigates the formation of stress-induced aggregates of TDP-43 in the cytoplasm [141]. Altogether, these data suggest that PARP1 plays a central role in the formation of stress granules and, therefore, in the pathogenesis of TDP-43-dependent ALS [140,141]. Interestingly, other PARPs have a clear role in the pathophysiology of TDP-43-associated ALS in addition to PARP1; *Drosophila melanogaster* Tankyrase-1 and Tankyrase-2 (also known as PARP-5a and PARP-5b; refer to §6 for details) regulate the specific cytoplasmic aggregation of TDP-43. Importantly, contrary to PARP1, the inhibition of tankyrases does not alter the overall formation of SGs [142]. Thus, the differential impact of PARPs' inhibition on formation of SGs suggests that PARP1 is deputed to the overall control of SG formation, while tankyrase activity is specifically required for TDP-43 nuclear-SG translocation. Through a PAR-binding motif in its N-terminal nuclear localization sequence, TDP-43 non-covalently binds PAR. The binding of TDP-43 to PAR leads to liquid–liquid phase separation of protein, which is required for its accumulation in stress granules. Downregulation of tankyrases and inhibition of PARP catalytic activity by using small-molecules reduces the accumulation of TDP-43 in the cytoplasm and potently mitigates neurodegeneration [142].

Altogether, all studies summarized above uncover a common role for PAR in the regulation of the subcellular re-distribution of proteins in response to cellular stresses and, eventually, in their cytosolic aggregation. Thus, the inhibition of PARPs’ functions can be considered a therapeutic strategy for neurological disorders that are characterized by PAR-dependent protein aggregation. Inhibitors of PARP1 activity possessing significant brain penetration are already commercially available, such as Pamiparib (BeiGene/Merck Serono) [10]. Nevertheless, new drugs may be required to specifically treat certain disorders that show different pathogenic mechanisms (e.g. XRCC1-dependent ataxias) [109].

### 4.3. PARP1 in inflammation-induced colorectal cancer

It is worth mentioning the contribution of PARP1 as well as of other ADPr players (see §§7 and 8) to the pathogenesis of inflammatory bowel disease. Colitis refers to inflammation of the inner lining of the colon. There are numerous causes of colitis including infection, ischaemia and allergic reactions. The inflammatory bowel diseases, Crohn's disease (CD) and ulcerative colitis (UC), are chronic inflammatory disorders of the gastrointestinal tract of unknown aetiology. The diseases are thought to be the result of a dysregulated mucosal immune response to commensal gut flora in genetically susceptible individuals [143–147]. Importantly, the association between long-standing and extensive colitis and an increased risk of colorectal cancer (CRC) is well established [148–150].

PARP1 plays crucial roles in both colon inflammation and CRC. By using protocols of carcinogenesis in animal models, it has been shown that PARP1 is required to protect against DNA alkylation and oxidation damage during the initial steps of CRC carcinogenesis. Consistent with this, PARP1-deficient mice challenged with alkylating drugs show high levels of DNA strand breaks compared with control animals, thus, confirming that PARP1 works as a caretaker tumour suppressor gene [151]. In addition, PARP1 promotes tumour growth by supporting the focal inflammation during the tumour progression. Indeed, PARP1-deficient mice show an attenuated innate immune response. The pro-inflammatory functions of PARP1 pass through the modulation of NF-κB activity and following activation of the IL6-STAT3-Cyclin D1 axis. Importantly, tissue microarray analyses reveal that *PARP1* is overexpressed in human CRC and its expression levels correlate with disease progression [151].

## 5. Macrodomain-containing PARPs in human disease

Twelve macrodomain-containing proteins are encoded in the human genome, including the previously mentioned hydrolase enzymes [3,42,68,69]. Three understudied PARP members are equipped with a number of macrodomains in addition to the PARP catalytic protein fold; thus, they are named Macro-PARPs [3,11,68]. Macro-PARPs were originally identified as members of a B-aggressive lymphoma protein family, which includes PARP9 (B-aggressive lymphoma 1; BAL1, also called ARTD9), PARP14 (BAL2, also called ARTD8) and PARP15 (BAL3, also called ARTD7) [11].

*PARP9* (*BAL1*) was identified in a genome-wide search for risk-related genes in chemo-resistant diffuse large B-cell lymphoma (DLBCL), the most common non-Hodgkin lymphoma. *PARP9* is largely overexpressed in DLBCL and promotes cell migration [11,12]. *PARP9* is also overexpressed in breast cancer [13]. At the molecular level, PARP9 plays roles in DNA damage repair. In response to DNA damaging agents, PARP9 localizes at the DNA damage foci via its macrodomain, which drives PARP9 at the PARP1-generated PAR foci. There, PARP9 interacts with the E3 ligase DTX3L (also known as B lymphoma- and BAL-associated protein; BBAP) and promotes DNA damage repair via the ubiquitination-dependent recruitment of BRCA1 (Breast Cancer Type 1 susceptibility protein), 53BP1 (p53-binding protein 1) and RAP80 (receptor-associated protein 80) [152]. PARP9 activity negatively regulates the function of PARP9/DTX3 L heterodimer complex by transferring single units of ADP-ribose specifically on the carboxyl terminal of glycine 76 of ubiquitin molecules, thus interfering with the canonical protein ubiquitylation system [153]. The oncogenic potential of PARP9 has been described to be dependent on its transcriptional functions, particularly required for IFNγ-mediated host inflammatory response. PARP9, whose expression is activated by IFNγ, interacts with the IFNγ receptor complex and STAT1 acting as a transcriptional co-repressor of anti-proliferative and pro-apoptotic genes, and as a co-activator for the transcription of responsive proto-oncogenes, such as IRF2 and B-cell CLL/lymphoma 6 (BCL6) [154,155].

PARP14 (BAL2) was initially identified as an interactor and transcriptional collaborator for Signal Transducer and Activator of Transcription 6 (STAT6) and, therefore, named Co-activator of STAT6 (CoaSt6) [156]. PARP14 plays roles mainly in transcription of interleukin-4 (IL4)-responsive genes, which control cell survival, metabolism and proliferation [14]. Under non-stimulating conditions, PARP14 binds the transcriptional repressors histone deacetylase 2 (HDAC2) and HDAC3 at IL4-responsive promoters [16]. Under IL4 stimulation, PARP14 ADP-ribosylates HDAC 2 and 3 leading to their dissociation and the recruitment of transcriptional co-activators including the p100 cofactor, which is also a substrate of PARP14 [16,157]. This process leads to the transcription of IL4-responsive genes, which are vital for both B and T cells. In B cells, PARP14-dependent transcription of IL4-responsive genes transduces pro-survival and anti-apoptotic signals [14]. In addition, by regulating the binding of STAT6 to the *Gata3* promoter, PARP14 and its enzyme activity are required for differentiation of T cells towards a T helper type-2 (Th2) lineage [158]. Th2 cells and Th2 cytokines (e.g. IL4, 5 and 13) associate with the promotion of IgE and eosinophilic responses and play a central role in the response to allergens, therefore, Th2 are the initiators of the allergic asthmatic condition [159]. Inhibition of PARP14 attenuates allergic airway disease, and it has been proposed as therapeutic strategy for asthma [158].

In addition to the transcriptional functions, PARP14 plays crucial roles in the metabolic control of cancer cells. PARP14 is indeed involved in the control of the cytokine-regulated glycolysis and glucose oxidation, thus, aiding the B-lymphoid oncogenesis [160]. Studies on solid tumours, such as sarcoma and hepatocarcinoma, further corroborate the link between PARP14 and cellular metabolism. In sarcoma cancer cells, PARP14 was shown to stabilize the glycolytic enzymes phosphoglucose isomerase (PGI) [15]. When secreted into the extracellular environment, PGI acts as a cytokine eliciting motogenic and differentiation cellular responses and, in addition, facilitates angiogenesis, metastasis and vessel leakiness [161–163]. In hepatocellular carcinoma, PARP14 inhibits JNK1-dependent phosphorylation and activation of the pyruvate kinase M2 isoform (PKM2), thus, promoting the aerobic glycolysis (Warburg effect) of cancer cells [17].

Lastly, PARP14 is involved in PARP10-dependent intracellular signalling. PARP14 binds MARylated proteins with high-affinity through its macrodomains. Among the ADPr proteins, PARP14 binds very efficiently automodified PARP10 and MARylated substrates of PARP10, such as the small GTPase RAN and the component of the NF-κB signal transduction pathway NEMO [164].

## 6. Role of tankyrases in the pathogenesis of cherubism

Tankyrase-1 and Tankyrase-2 are PARP enzymes characterized by large ankyrin repeating domains. Tankyrases play roles in telomere length maintenance which is particularly relevant for ageing, homologous recombination-mediated DNA damage response, mitosis, pexophagy, and Wnt- and Notch-mediated signal transduction [165–174].

Differently from other PARPs, tankyrases engage their protein substrates through the ankyrin domains within their protein sequence that bind very efficiently a well-defined octapeptide consensus within protein substrates [175]. The consensus for binding to tankyrase proteins consists of arginine in position 1, small and hydrophobic residue in position 4, aspartate in position 5, however, glutamic acid, valine, glutamine, tyrosine, isoleucine and cysteine are equally tolerated, and glycine in position 6 [175]. Protein–protein interaction is therefore the prerequisite for tankyrase-dependent PARylation. A large number of binders/substrates of tankyrase proteins have been proposed by interaction studies (608 proteins) [173,175]. Among tankyrase-interacting proteins there are AXIN1/2, the telomeric-repeat binding factor-1 (TRF1), the insulin-responsive amino-peptidase (IRAP), the 182-kDa tankyrase-binding protein (TAB182), the nuclear mitotic apparatus protein-1 (NuMA1), 3BP2, Notch2, HectD1, NKD1 and NKD2, the CBP80/CBP20-dependent translation initiation factor (CTIF), BLZF1 (basic leucine zipper factor 1), CASC3 (cancer susceptibility factor 3), the component of the HIPPO signalling pathways AMOT (Angiomotin) and PTEN, although it is not clear whether the latter is a direct tankyrase-binding protein or substrate [165,168,170,173,176–180]. Following the interaction and PARylation, a large portion of tankyrase-binding proteins become targets of proteasome degradation [175]. Indeed, tankyrase-mediated PARylation of protein substrates acts as a scaffold for recruitment of the PAR binding motif-containing protein RNF146, an E3 ubiquitin ligase. Thus, RNF146 binds tankyrases' PARylated substrates and ubiquitinates them, leading to their proteasome degradation. The liaison between tankyrase-mediated ADPr and proteasome degradation was initially observed in the regulation of the proliferative WNT pathway in CRC cells through the modification and following degradation of AXIN1/2, then expanded to many other cellular processes regulated by tankyrase substrates [10,170,181]. Thereby, the interest in targeting tankyrase proteins for the pharmacological modulation of pathological conditions has increased in recent times. In particular, the involvement of tankyrases in Wnt signalling (related to tumourigenesis) and glucose homeostasis (related to diabetes) promises advances for targeting tankyrases for therapeutic interventions, as demonstrated by the pre-clinical experimentation of tankyrase inhibitors for treatment of CRC [10,170,181–186]. In addition, the chemical inhibition of tankyrase proteins has been proposed for treatment of brain injuries of the newborn. Indeed, tankyrase small inhibitors stabilize Axin2 levels in oligodendrocyte progenitor cells from brain and spinal cord, thus accelerating differentiation and myelination after hypoxic and demyelinating injury [187].

Dysregulation of tankyrase-mediated binding and degradation of protein substrates has been recognized as the pathogenic mechanism of cherubism, a dominantly inherited human disorder. Cherubism is a bone inflammatory destructive disease characterized by deformities of the facial bones [188]. Cherubism is caused by single missense mutations in Sh3bp2, the gene that encodes the adaptor protein 3BP2 [189]. Most 3BP2 mutations associated with cherubism cluster within the peptide sequence RSPPDG, such as R413Q, P416H, or G418R mutations, which serve as a tankyrase-interacting motif. Similar to all known targets of tankyrase, PARylation of 3BP2 leads to its proteasome degradation, which is required for controlling 3BP2 protein levels within the cells. In cherubism, 3BP2 mutations in the RSPPDG hexapeptide impair tankyrase-mediated protein degradation, which in turn translates into elevated steady-state protein levels of 3BP2 in primary cells deputed to maintain the bone homeostasis, namely osteoclasts. As a result of this dysregulation, the signalling pathway including SRC, SYK and VAV proteins is up-regulated leading to uncontrolled activation of osteoclasts’ functions and peculiar interosseous fibrocystic lesions in cherubism-affected patients [175,190].

## 7. Role of ADP-ribosyl hydrolases in human disease

### 7.1. Macrod1 and MacroD2

MacroD1 and MacroD2 are related mono(ADP-ribosyl) hydrolases belonging to a subfamily of proteins present in both eukaryotes and prokaryotes [191]. MacroD1 and D2 contain nearly identical catalytic macrodomains that, by using substrate-assisted catalysis, hydrolyse the ester bond joining the ADP-ribose to the acidic residues of acceptor proteins or cleaving OADPR [74,192]. However, MacroD1 and D2 cannot hydrolyse the *O*-glycosidic bond of Ser-ADPr [66] (figure 1).

MacroD1 (also named Leukaemia-Related Protein 16; LRP16) contains a leading sequence localizing the protein at the mitochondria; nevertheless, its roles in transcription as a cofactor for androgen and oestrogen receptors and in NF-κB signal transduction cascade have been largely established [193–196].

MacroD1 appears overexpressed in several human cancers (such as endometrial carcinoma, gastric carcinoma, CRC and breast carcinoma) and its expression levels correlate with poor prognostic outcomes [25–30]. It is worth mentioning that the oncogenic potential of MacroD1 in CRC depends on its ability to activate the pro-survival NF-κB-dependent signalling in the presence of DNA damage. When stimulated by DNA-damaging agents, MacroD1 enriches in the cytosol of CRC cells where it interacts with double-stranded RNA-dependent kinase (PKR), thus facilitating the kinase activation, and promoting the binding with IKKβ. The formation of MacroD1/PKR/IKKβ ternary complex triggers the activation of anti-apoptotic signals mediated by NF-κB. Importantly, a screening of molecules targeting MacroD1 macrodomain led to the identification of a small molecule (MRS2578) that, both *in vitro* and *in vivo*, abrogates MacroD1- and NF-κB-dependent pro-survival signals synergistically with DNA-damaging chemotherapies [28].

The other member of the MacroD subfamily, MacroD2, also shows connections with the NF-κB pathway. MacroD2 was shown to play a central role in reverting PARP10-dependent MARylation of protein substrates, as in the case of GSK3*β* kinase, a kinase involved in the WNT pathway. Additionally, MacroD2 may revert PARP10-dependent MARylation of NEMO (NF-κB essential modulator), modification of which results in reduced NEMO polyubiquitylation and decreased NF-κB signalling [68,75,197,198].

In disease, MacroD2 shows association with neurological disorders, such as autism and kabuki syndrome (KS), as well as cancer.

Autism is a heterogeneous neurodevelopmental disorder defined by deficits in language and social behaviour, as well as patterns of repetitive behaviours of high heritability [199,200]. However, a simpler genetic basis for autistic or autistic-like traits is recognizable in around 5% of autism individuals with diseases [201]. Single-nucleotide polymorphisms (SNPs) associated with autism are found in few candidate genes, among them the *MacroD2* gene [31–33]. kabuki (or Niikawa-Kuroki) syndrome is a genetically heterogeneous dominant mental retardation with a described autosomal transmission. It is characterized by postnatal growth retardation, typical facial defects, fetal pads, cleft palate and major malformations of the heart, kidneys and vertebra [202]. A mutation screening revealed a 250 kilobase *de novo* microdeletion at 20p12.1, which hits intron 5 of *MACROD2* gene and associates in one patient with kabuki syndrome [34]. An intron 5 deletion of the *MACROD2* gene was also reported in a patient displaying a kabuki-like phenotype [35]. It is worth mentioning that the association between an intron deletion of *MACROD2* gene and kabuki syndrome is reported in a small number of clinical cases. Thus, more research is needed to clarify the specific link between *MACROD2* and KS [203]. Genome-wide DNA copy-number analyses across human cancers have indeed revealed that common focal deletions of *MACROD2* genomic locus happen in multiple malignancies, such as in stomach adenocarcinoma, cervical squamous cell carcinoma and endocervical adenocarcinoma, esophageal carcinoma, uterine corpus endometrial carcinoma, uterine carcinosarcoma, lung adenocarcinoma, liver hepatocellular carcinoma and thyroid carcinoma [36,37]. Importantly, some *MACROD2* somatic mutations are found in CRC. Loss of variable size of the genomic locus containing *MACROD2* as well as missense mutations is quite frequent in CRC. Importantly, some *MACROD2* somatic mutations observed in cancer are predicted to interfere with binding of ADP-ribose to the catalytic pocket of MacroD2, therefore leading to increased sensitivity to genotoxic stress, and chromosomal instability in CRC [38].

### 7.2. Terminal ADP-ribose glycosylhydrolase 1 (TARG1)

TARG1 (c6orf130) is a macrodomain-containing protein with similar substrate specificities as seen for MacroD1 and MacroD2—it can cleave glutamate-linked protein ADPr, OADPR and phosphate-linked ADPr on nucleic acids [73,75,204,205] (figure 1). Nevertheless, the macrodomain of TARG1 is very diverged from those in PARG and both MacroD1 and MacroD2 proteins, and adopts a distinct catalytic mechanism [39].

A distinct homozygous sequence variant of the *TARG1* gene was found in a family with a number of members affected by a severe and progressive neurodegeneration and seizure disorders. The sequence variant associated with disease is characterized by a premature stop codon within the exon 4 of *TARG1* locus and predicts the formation of a truncated and not functional TARG1 enzyme. Importantly, *TARG1* knockdown in human cells leads to significant proliferation defects and sensitivity to DNA damage [39].

Interestingly, the phenotype of *TARG1*-mutated patients somewhat resembles a clinical case described in the early 1980s of an 8-year-old male who died after a 6-year course of progressive neurologic degeneration and renal failure. Biochemical studies performed on bioptic specimens obtained from this patient showed the lysosomal accumulation of glutamyl ribose 5-phosphate (a glutamate amino acid linked to a phosphoribose group), which was proposed to arise from the inability to cleave the glutamate-linked ADPr on proteins. However, the identity of the deficient gene remained uncovered [206,207]. The accumulation of peptides linked to a phosphoribose group (phosphoribosylated peptides) suggests the presence of alternative hydrolytic mechanisms in cells that allow cleavage of the phosphodiester bond within MAR or PAR attached to a protein. Such pathways could intervene both under physiological and pathological conditions; for instance, when not functional hydrolytic enzymes (e.g. in the case of TARG1) lead to an excess and toxic accumulation of MARylated and PARylated proteins. Interestingly, specific members of two unrelated classes of phosphodiesterases were shown to possess ability to produce protein phosphoribosylation *in vitro*, the nucleoside diphosphates linked to X (any moiety) (NUDIX) and ectonucleotide pyrophosphatase/phosphodiesterase (ENPP) [208–210].

### 7.3. Poly(ADP-ribosyl)glycohydrolase (PARG)

PARG contains a highly diverged macrodomain fold and its structure has been extensively studied [73,211–214]. Distinctly from TARG1, MacroD1 and MacroD2, PARG has an insertion of a unique catalytic loop in the conserved globular macrodomain fold, which contains the catalytic residues and is essential for degradation of PAR chains. PARG preferably binds PAR at the chain termini and sequentially degrades ADP-ribose units (exo-glycohydrolase activity; figure 1). The endo-glycohydrolytic cleavage of PAR chains is also catalysed by PARG, but this activity is less efficient [213,215,216]. Yet PARG endo-glycohydrolase activity may become significant in the presence of excessive PAR production, for instance in cells or tissues exposed to abundant oxidative stress as observed in neurological disorders caused by aggregation of cytotoxic proteins (e.g. in PD) [110]. Indeed, the release of free long PAR fragments was shown to trigger apoptotic signalling [217].

Although genetic mutations have not been directly linked to human diseases, PARG cellular functions may significantly contribute to the pathogenesis of hereditary and acquired disorders. Contrary to PARP1 inhibition or deletion, which is not lethal for cells and mice (although it increases radiosensitivity), *PARG* knockout results in embryonic lethality in mouse model as a result of PAR accumulation and cellular apoptosis. However, *Parg* null mouse trophoblast-derived stem cells can be successfully cultivated in the presence of PARP inhibitors, suggesting that PARP1 inactivation can rescue *PARG* deletion [218]. Similarly, *Parg* null *Drosophila melanogaster* flies die at the embryonic stage; however, when grown at a permissive temperature, survival is increased. The surviving flies display PAR accumulation, neurodegeneration, reduced locomotion and premature death [219]. In line with data obtained in the *D. melanogaster* model, depletion of nuclear *PARG* isoforms in mice results in PAR accumulation in the brain [220]. Altogether, the body of information provided by *PARG*-deficient models further supports the essential role of PAR in the regulation of cellular homeostasis, especially in neuronal cells.

PARG functions have also been linked with the pathogenesis of inflammatory and neoplastic disorders. As for PARP1, a murine experimental model of colitis shows the contribution of PARG in sustaining the inflammatory response in the colon. Mice harbouring a deletion of the 110-kDa isoform of PARG protein, which are viable and fertile, are resistant to colon injury when challenged by dinitrobenzene sulfonic acid (DNBS) and show an attenuated inflammatory response [221].

According to experimental models of colitis, the serum titre of antibodies against PARG is a marker of mucosal damage caused by refractory ulcerative colitis [222].

Database analysis of sequencing data from The Cancer Genome Atlas (TCGA) revealed that *PARG* is overexpressed in many tumour types, in particular in breast tumour tissues, where it appears to be approximately fivefold more expressed than in normal epithelium. Approximately 15% of all invasive ductal breast tumours showed elevated *PARG* mRNA level, with the frequency reaching the 20% in HER2-positive and triple-negative subtypes. Thus, PARG levels are associated with a poor prognosis in breast cancers. Depletion of *PARG* significantly impairs the growth and metastasis of triple-negative breast tumours, in both *in vitro* and *in vivo* models, thus highlighting the therapeutic potential of PARG inhibition in breast cancer [223]. Importantly, the inhibition of PARG has already been proposed as a therapeutic treatment of human cancers [10,224]. This would be particularly appropriate for the treatment of aggressive breast cancers. It is worth mentioning that *PARG* inactivation often occurs as a resistance mechanism to PARP inhibitors in human serous ovarian and triple-negative breast cancers. Indeed, the genetic loss of *PARG* restores PAR formation and partially rescues PARP1 signalling [225].

### 7.4. ADP-ribosyl-acceptor hydrolase 1 (ARH1)

ARH1 is a cytosolic and ubiquitously expressed protein. Although the structure and mechanism are highly similar to ARH3 [63], ARH1 possesses a robust mono-ADP-ribosyl hydrolytic activity towards *N*-glycosidic bonds of arginine-modified proteins, and no activity against Ser-ADPr [48,62,63,226,227] (figure 1). By using both *in vitro* and *in vivo* models, it was shown that ARH1 plays a role in tumour genesis and progression. Indeed, *Arh1*-deficient mice spontaneously develop multiple malignancies, including lymphoma, hepatocellular carcinoma and hemangio-/rhabdomyosarcoma [228]. Studies performed in *Arh1* heterozygous mice and in nude mice injected with *Arh1*-null MEFs showed the loss of heterozygosity (LOH) of the remaining *Arh1* allele or loss of *Arh1* gene activity due to spontaneous mutagenesis. Genome sequencing of mice revealed that *Arh1* gene mutations were located in exons encoding the catalytic site. Analysis of human cancer COSMIC database revealed 32 *ARH1* mutations found in human lung, breast and colon cancers; 70% of those mutations were missense mutations with single-base substitution, which surprisingly overlap with the mutations that spontaneously generate in *Arh1* heterozygous mice [21]. Among those mutations, the D56N hits one of the two conserved aspartates (positions 60 and 61 in mouse Arh1) that are required for Mg^2+^ coordination and hydrolase activity [63,229].

### 7.5. ADP-ribosyl-acceptor hydrolase 3 (ARH3)

ARH3 was initially identified as a back-up PAR-degrading enzyme. Similar to PARG, ARH3 primarily cleaves the chains as exo-glycohydrolase, however, its specific activity against long PAR chains is nearly two levels of magnitude lower than for PARG [62,63,66,230]. Later on, ARH3 was shown to be the main hydrolase responsible for cleaving the ADPr from modified serine residues [66,231] (figure 1). The catalytic fold of ARH3 is completely different compared with PARG, which is instead a macrodomain-containing protein. In turn, the structural divergence reflects in a different conformation of ADP-ribose within the catalytic pocket as well as in a different catalytic mechanism [63,232–234]. As for ARH1, the presence of conserved aspartates (D77 and D78 in human ARH3), are essential for coordination of Mg^2+^ within ARH hydrolase [229].

At the cellular level, most of the ARH3 is found in cytoplasm, nucleus and mitochondria. The mitochondrial localization of ARH3 is determined by the presence of a mitochondrial-targeting sequence at the N-terminus, which suggests a role for ARH3 for ADPr degradation in mitochondria [230,235]. Nevertheless, all the ARH3 cellular functions described so far seem to converge on safeguarding genome stability. As discussed above, Ser-ADPr is the most abundant type of ADPr modification in response to genotoxic stress and it can be reversed only by ARH3, as far as we know. ARH3 is indeed able to cleave the terminal *O*-glycosidic bond joining the ADP-ribose and the serine of modified protein substrates [66,85]. ARH3 was also shown to act on free oligomers of PAR in cells released upon PARG endoglycohydrolase activity [236,237]. By doing so, ARH3 may control a mechanism of PARP1/PAR/AIF-mediated cell death (also known as Parthanatos) [236]. Altogether these data support the hypothesis that ARH3 could be involved in the pathogenesis of human disorders characterized by the cytotoxic and pro-apoptotic accumulation of PAR, such as in neurological disorders (e.g. in PD and AD).

Importantly, autosomal-recessive inherited genetic variants of ARH3 are directly linked with neurodegenerative disorders. Two independent studies have described 28 individuals belonging to fourteen families, which associate recessive and inactivating *ARH3* gene mutations with paediatric-onset neurodegenerative disorder characterized by brain atrophy, developmental delay or regression, seizures, infection-associated episodes of ataxia, and axonal sensori-motor neuropathy [22,23]. It should be noted that most of the detected truncations/mutations predictably affect protein stability. As expected, ARH3 deficiencies associate with the accumulation of cellular ADPr, which drastically affects cell viability. Both the cellular accumulation of ADPr and the following cell death are prevented by treatment with PARP inhibitors [22,23]. Thus, these results propose once again the inhibition of PARP1 as a therapeutic strategy for the treatment of neurodegenerative diseases.

It is worth mentioning that, although all *ARH3* patients show overall overlapping clinical features, both studies have not established an obvious genotype–phenotype correlation, for instance, regarding the onset and additional complications of the disorder. This observation suggests that additional factors, such as the genetic background or the exposure to environmental challenges, may contribute to the phenotypic variability among individuals. Considering the crucial roles of ARH3 in response to cellular stresses (e.g. oxidative and DNA damage insults), the exposure to stress conditions may be particularly important to anticipate the onset or worsen the neurodegenerative traits of disease.

## 8. Additional macrodomain-containing proteins in human disease

### 8.1. GDAP2 (MacroD3)

*GDAP2* gene (found in metazoans and plants) encodes an uncharacterized additional macrodomain-containing protein, which has been recently linked to a human hereditary disorder characterized by ataxia, progressive spasticity and dementia [24]. Although GDAP2 macrodomain is similar to the one of MacroD1/2 proteins, it does not seem to bind derivatives of ADP-ribose, but instead, it appears to possess some affinity for poly(A) [69,72]. Yet the pathogenic mechanisms underlying GDAP2 deficiency remain unclear.

### 8.2. ALC1 (CHD1L)

The human ALC1 (amplified in liver cancer 1; also known as CHD1L (chromodomain-helicase-DNA-binding protein 1-like) gene encodes a member of the SNF2 (sucrose non-fermenter 2) superfamily of ATPases. Among SNF2 family members, ALC1 is unique because it includes a macrodomain that is capable of binding PAR. The binding of ALC1 to activated and PARylated PARP1 is crucial, but not sufficient, for DNA-dependent ATPase and ATP-dependent nucleosome remodelling activities [238–243].

ALC1 was originally identified as a gene amplified in hepatocellular carcinomas [18]. Overexpression of the ALC1 protein was found to transform human cells and to be oncogenic in mice [18,244,245]. A role for the oncogene ALC1 has also been demonstrated in breast and CRC [19,20].

In addition, gene mutations in human *ALC1* were found in patients affected by congenital anomalies of the kidney and urinary tract [246].

## 9. ADPr and infectious disease

From the perspective of human pathologies bacterial ADPr systems can roughly be divided into two groups: the secreted exotoxins, which participate directly in promoting bacterial infection and associated symptoms; and those that have an internal role in bacterial stress-response. The latter potentially have major functions in persistence and have been proposed as potential therapeutic targets.

The exotoxins group encompasses a variety of bacterial ADP-ribosyl transferase toxins (bARTTs). MARylation of eukaryotic targets by bARTT is usually irreversible and aims at nucleotide-binding proteins, prevalently GTP- and, in some cases, ATP-binding proteins [44]. Interestingly, the deficiency of human ARH1 hydrolase leads to an increased sensitivity to cholera toxin, suggesting that bacterial ADPr can be reversed by the host hydrolases [65].

As outlined in §2 of this review, two subfamilies of bARTTs can be distinguished based on their structure and target proteins: diphtheria-like and cholera-like toxins, the latter encompassing an additional two subgroups, C2-like binary and C3-like toxins.

Diphtheria toxin ADP-ribosylates the eukaryotic elongation factor 2 (EF2), a GTP-binding protein essential for protein synthesis in the cell. The modification halts the entire protein synthesis and, in turn, leads to cell death [44]. The same mechanism of action is shared by the Exotoxin A from *Pseudomonas aeruginosa*, a ubiquitous multidrug-resistant pathogen [247].

Cholera and cholera-toxin-like proteins (e.g. the heat-labile enterotoxin from *Escherichia coli* and the pertussis toxin from *Bordetella pertussis*) transfer ADP-ribose onto heterotrimeric G proteins. The modification locks the subunit α of G proteins in a GTP-bound state, which constitutively stimulates host adenylate cyclase. In the case of cholera and enterotoxin, constitutive activation of G proteins results in opening and efflux of the chloride ions together with water [44]. Pertussis toxin acts towards virtually all mammalian cell types and has a broad array of effects on host cell activities [248]. ADP-ribosyl transferase subunit of typhoid toxin from *Salmonella typhi* (exclusively human pathogen) is structurally similar to pertussis toxin; however, the pathogenic mechanisms as well as the proteins substrate(s) of this toxin remain unknown [249].

The C2-like toxins from *Clostridium* sp*.* [44,250] and the newly characterized SpvB from *Salmonella* sp*.* [251] are examples of toxins ADP-ribosylating non-polymerized form of actin. The MARylated G-actin, upon incorporation into filaments, inhibits further integrations resulting in serious impairments of cellular cytoskeleton.

The C3-like toxins expressed by *Clostridium botulinum*, *Bacillus cereus*, *Staphylococcus aureus* and others target small Rho GTPase enzymes, which modulate actin polymerization. The MARylation of Rho GTPase alters the interaction with protein partners, thus locking themselves in a deactivated state. The consequences are similar to that of the C2-like toxins—the disintegration of the cytoskeleton. The recently described SpyA from *Streptococcus pyogenes* targets another cytoskeletal protein-vimentin, and actin to a lesser degree [252].

In addition, a very intriguing class of bARTTs has been described in *Legionella pneumophila*. The *Legionella* protein SdeA modifies ubiquitin molecules of the host by transferring ADPr on arginine 42, thus impairing the physiological ubiquitination processes. Through a process of phosphoribosyl-ubiquitination, MARylated ubiquitin is in turn transferred onto serine residues of protein substrates, therefore modulating the endogenous functions of modified proteins, such as Rab33 [253–255]. This ADPr system is reversible, as it can be counteracted by another bacterial protein, SidJ, acting as a hydrolase [256].

One of the best-studied stress-response systems in bacteria is the toxin-antitoxin (TA) module. There are more than 1000 TA modules known [257]. Among them, the only known module to exploit the ADPr system is the DarT/DarG module, which is found in various bacteria, including the global pathogen *Mycobacterium tuberculosis* [258]. DarT is an ART able to MARylate the single-stranded DNA on specific thymidine residues, which impairs cellular processes essential for bacterial growth and activates SOS response. The macrodomain protein DarG, which hydrolyses the ADP-ribosylated DNA, counteracts DarT activity [258].

Another example of ADPr system in bacterial stress response is operated by sirtuins. While the mammalian sirtuins seem to act primarily as NAD-dependent deacetylases, a diverged class of sirtuins present in pathogenic bacteria and fungi (called SirTMs) exhibits a robust protein ADPr activity that is regulated by another protein modification: lipoylation. This mechanism was shown to modulate the microbial oxidative stress response [259].

## 10. ADPr in viral infections

Viruses from the *Coronaviridae*, *Togaviridae* and *Hepeviridae* families all contain genes encoding macrodomain-containing proteins, suggesting a role for ADPr during infection diseases [260–265]. Notably, several human PARPs have been shown to be activated and function in the host antiviral response. For instance, PARPs 9, 12, 13 and 14 are among the 62 Interferon-stimulated genes and overexpression of PARPs 7, 10 or 12 inhibits alphavirus replication [266]. In addition, PARPs 5a, 12, 13, 14 and 15 localize at the stress granules, well-known cytoplasmic structures with antiviral functions; interestingly, the integrity of stress granules is inhibited by the alphaviral macrodomain-containing nsP3 [138,267]. Thus, it appears that ADPr is required for a proper host antiviral response and that viruses have evolved systems (mainly consisting of macrodomain-containing proteins) able to modulate defensive host ADPr systems. Not surprisingly, PARPs 4, 9, 13, 14 and 15 show a rapid evolution as a result of a strong recurrent positive selection in the attempt to escape the modulation operated by viral proteins [268,269].

## 11. Concluding remarks

Numerous pioneering findings have shown the impact of ADPr on many vital cellular processes, the dysregulation of which is known to lead to human disorders. Many genes involved in ADPr are now known to be mutated or dysregulated in various acquired and hereditary diseases, such as neurological disorders and cancer. By contrast, the pharmacological modulation of ADPr by small-molecule inhibitors can be a potent tool to treat human diseases. Research within the ADPr field has been progressing particularly fast in recent years, and it is hoped that this will provide new avenues for the therapeutic interventions.

## Supplementary Material

Reviewer comments

## Data accessibility

This article has no additional data.
