# Supplementary material for: ADP-ribosylation signalling and human disease
Source: Open Biol. 2019 Apr 17;9(4):190041. doi: 10.1098/rsob.190041 (PMC6501648; doi:10.1098/rsob.190041)
Supplement: Reviewer comments [file rsob190041_review_history.pdf]

THE ROYAL SOCIETY
PUBLISHING

OPEN
BIOLOGY

ADP-ribosylation signalling and human disease

Luca Palazzo, Petra Mikolčević, Andreja Mikoč and Ivan Ahel

Article citation details
Open Biol. **9**: 190041.
http://dx.doi.org/10.1098/rsob.190041

Review timeline
Original submission: 13 February 2019
Revised submission: 20 March 2019
Final acceptance: 22 March 2019

Note: Reports are unedited and appear as submitted by the referee. The review history appears in chronological order.

Review History

RSOB-19-0041.R0 (Original submission)

Review form: Reviewer 1

Recommendation
Accept with minor revision (please list in comments)

Are each of the following suitable for general readers?

a) **Title**
Yes

b) **Summary**
Yes

c) **Introduction**
Yes

Is the length of the paper justified?
Yes

Reports © 2019 The Reviewers; Decision Letters © 2019 The Reviewers and Editors; Responses © 2019 The Reviewers, Editors and Authors. Published by the Royal Society under the terms of the Creative Commons Attribution License http://creativecommons.org/licenses/by/4.0/, which permits unrestricted use, provided the original author and source are credited

Should the paper be seen by a specialist statistical reviewer?
No

Is it clear how to make all supporting data available?
Not Applicable

Is the supplementary material necessary; and if so is it adequate and clear?
Not Applicable

Do you have any ethical concerns with this paper?
No

Comments to the Author
The review by Palazzo et al. details the current knowledge on the disease connections of ADP-ribosylation signaling. The review is well written, easy to understand and provides a valuable source of the scientific reports that establish the role of ADP-ribosylation in various human diseases.
The authors do not specifically spell out but the review focuses on the enzymes involved in ADP-ribosylation metabolism. There is probably only one exception: GDAP2, which has not been associated with either generating or degrading ADP-ribose. The 7.4 GDAP2 section is quite short and I wonder if other ADP-ribose readers associated with disease could be mentioned there, such as the macro histones and Alc1.
Regarding disease and in particular the hope of alleviating it, the therapeutic potential through targeting the players of ADP-ribosylation signaling could be discussed. Especially the review of the current status of developing selective small-molecules and the potential challenges based on the known structures and available inhibitors of the enzymes to be targeted would be a nice addition.
A table detailing the proteins and the associated diseases with references would be a great help for the reader looking for specific questions.
In section 7.6, it is written that the fold and catalytic mechanism of ARH3 are completely different from PARG. While there are several original publications to support such statement, authors could briefly mention some information they find important, such as how that translates to similarities and differences in function or the potential for their therapeutic targeting.

Minor comments on typos:
P2L19 "transferred onto on nucleic" omit "on"
P5L24 "The member 1 of the mammalian PARPs" does not read well, please rephrase.
P7L32 " characterized by; aggregation" omit semi-colon.
P8L16 "imbalance" might be better than "unbalance".
P8L32 "splice variants" instead of "splicing variants"
P9L25 Inflammatory bowel disease is a type of colitis, I suggest omitting "colitis".
P9L26 Not all mentioned are causes but also manifestations. Infection, ischemia and allergic reactions are causes the others not.
P10L4 "Alkylating" instead of "alkylation"
P10L33 Could authors rephrase "passing though"?
P12L9 and L12 Delete "s" from "plays" and "proteins"
P12L27 Delete the surplus "the"
P14L21 kilobase
P21L7 "hydrolyzes" instead of "hydrolases"

Review form: Reviewer 2

Recommendation
Accept with minor revision (please list in comments)

Are each of the following suitable for general readers?

a) **Title**
Yes

b) **Summary**
Yes

c) **Introduction**
Yes

Is the length of the paper justified?
Yes

Should the paper be seen by a specialist statistical reviewer?
No

Is it clear how to make all supporting data available?
Not Applicable

Is the supplementary material necessary; and if so is it adequate and clear?
Not Applicable

Do you have any ethical concerns with this paper?
No

Comments to the Author
This excellent review will no doubt be well-received by anyone interested in a comprehensive yet concise summary of disease-relevant functions of ADP-ribosylation. The manuscript refers to numerous recent studies, covers a wide range of enzymes in different systems, including bacterial toxins/antitoxins and touches upon the function of ADP-ribosylation in viral infection. I enjoyed reading this unique manuscript and would be happy to see it published in Open Biology.

I have a number of suggestions for revising the manuscript.

Major points:
- The inhibition of DNA-dependent PARP enzymes (most notably PARP1) in BRCA-deficient cancers is not discussed. This is certainly one of the most tangible successes in targeting PARPs in the clinic. Mechanistic insights are being gained: trapped PARP1 on DNA breaks appear to be the toxic lesion initiating synthetic lethality. I think it is rather important to discuss this. If the authors would like to limit the discussion, they can refer the reader to other excellent reviews on the topic.
- The field suffers from a somewhat clumsy nomenclature, even referring to mono-ADP-ribosyltransferases as PARPs. (See also Figure 1: the term "mono-PARP" is contradictory in itself.) The review would be an opportunity to use a nomenclature that is clearer to the outsider. Many authors prefer the old, inconsistent nomenclature for historic reasons, but this may impair clarity for readers not yet familiar with the PARP field. This will be the authors' call.
- p.9, paragraph 1: There is an apparent contradition, or at least a potential source for

misunderstanding. In line 7, the authors state that "inhibition of Tankyrases does not alter the overall formation of SGs". In contrast, in lines 10 and 11, the authors point out that PARP inhibition "reduces the accumulation of TDP-43 ...". Are SGs still being observed?
- Two major incentives for targeting the tankyrase proteins by small molecule inhibitors are to control their function in canonical Wnt signalling (for example related to colorectal cancer) and glucose homeostasis (related to diabetes). Can the authors either briefly discuss these aspects or refer the reader to other reviews? Tankyrase has also gained attention through its roles in telomere length maintenance. A few more words on this aspect, which is also relevant to disease mechanisms, would be welcomed.
- I think the following study deserves to be discussed in the context of tankyrase, in particular since neurobiology already features prominently in the review: Fancy et al. (2011) Axin2 as regulatory and therapeutic target in newborn brain injury and remyelination. Nat. Neurosci. 14, 1009–1016.
- p. 12, paragraph 3: Tankyrase appears to impinge on a wide range of functions and have many interaction partners and substrates. The sheer number of binders/substrates, illustrated by references 160 and 150, should be referred to. Also, another study worth adding in this context is the following: Li et al. (2017) Proteomic Analysis of the Human Tankyrase Protein Interaction Network Reveals Its Role in Pexophagy. Cell Reports 20, 737–749.
- p. 12, line 8: Functions of tankyrase in HR-mediated DNA damage repair and Notch signalling are fairly recent discoveries. Therefore, it may be a bit premature to talk about "crucial roles". This may well be the case, but without a deeper mechanistic understanding, I recommend caution in the wording. Among the best understood functions of tankyrase are canonical Wnt signalling, telomere length maintenance and roles in mitosis.
- p. 12, line 13: Many tankryase-binding motifs in fact are octapeptides, as informed by peptide permutation studies (see reference 160). The consensus has also been determined in greater detail than RxxPDG (also see reference 160). Also, "non-canonical" motifs with an N-terminally displaced arginine residue are emerging (see DaRosa et al., Protein Sci, 2018 vol 27). Having said that, the particular motif in 3BP2 is indeed an example for a hexapeptide as the residue at position 8 makes minimal contributions to binding.
- p. 12, line 20: Whether PTEN is a direct tankyrase binder or substrate is still not clear.
- p. 12, paragraph 3: Although the PAR-dependent ubiquitination pathway is ubiquitous, many tankyrase binders do not become PARylated or destabilised (e.g., MCL1, GMD). The mechanistic basis for this observation is not known. The sentence in lines 21 and 22 should therefore be revised accordingly.

Minor points / stylistic suggestions / typos:
- The abbreviation ADPr is used for both ADP-ribosylation and ADP-ribose in the article. A disambiguation would be desirable.
- Abstract (p. 1): "The control of the ADPr network is vital, and dysregulation ..."
- Abstract (p. 1): "... acquired human diseases, such as several neuological disorders ..."
- p. 2, line 7: This is a conceptual point: cellular mechanisms do not evolve for a purpose; evolution is not purpose-directed.
- p. 2, line 8: "dynamically regulate protein function through chemical modification." - You do not need the word "structure" here.
- p. 2, line 32 and elsewhere (p.9, line 20): talking of "cure" is very strong. This could, for example, be exchanged by "available therapy".
- p. 3, line 27: "paragraph thirteen" - indicating the section would suffice as readers may not want to count paragraphs.
- p. 3, line 32: "extracellular"
- p. 6, line 9: "Ser-ADPr targets include ..."
- p. 6, line 10: "has been found linked to factors mediating RNA processing, chromatin modification, splicing, ..."
- p.6, line 26: "... which involves the production ..."

- p. 6, line 29: "Similar observatiuons were made ..."
- p. 6, line 30 & 31: "Moreover, PARP1 activation was shown in [...] patients who died of stroke, ..."
- p. 7, line 7: I suggest saying "We next describe the role of PARP1 activation ..."
- p. 7, line 32: "... which is characterized by aggregation ..."
- p.8, line 34: The sentence commencing with "In turn, nuclear PAR aids the translocation ..." is unclear. Can the mechanism be described somewhat more extensively?
- p. 10, line 14: "... are encoded in the human genome ..."
- p. 10, line 25: The sentence "PARP9 is recruited at the DNA damage foci ..." is unclear.
- p. 10, line 32: Likewise, the sentence "It should be noted that the oncogenic potential ..." can be written in a clearer way.
- p. 11, line 13: "... which also is a substrate of PARP14 ..."
- p. 11, line 28: "When secreted into the extracellular environment [...] and, in addition, facilitates angiogenesis ..."
- p. 12, line 28: "tankyrase substrates"
- p. 13, line 17: "OADPr" hasn't been defined.
- p. 14, line 17: "among them the MacroD2 gene"
- p. 14, line 21: "250 kilobase"
- p. 14, line 24: "It is worth mentioning ..."
- p. 14, line 25: I suggest separating the sentence and have the second sentence start with "Thus, more observations are needed to explore ..." (rather than "establish")
- p. 14, line 33: "Importantly, some MacroD2 somatic mutations ..."
- p. 15, line 7: "... phophate- [or phospho-]linked ADPr [...] [44, 47, 188, 189]"
- p. 15, line 24: The logic of the sentence "Nonetheless, the accumulation of peptides ..." is unclear.
- p. 16, line 2: "Differently from TARG1, MacroD1 and MacroD2, ..."
- p. 16, line 13: "... have not been directly linked ..."
- p. 16, line 20: "... die at the embryonic stage; however, when grown at a permissive temperature, survival is increased."
- p. 16, line 27: "PARG functions have also been linked ..."
- p. 17, line 10: "PARG has already been proposed ..."
- p. 17, line 10: I suggest splitting the sentence, starting the second sentence with "This would be particularly appropriate ..."
- p. 17, line 19: one reference is formatted incorrectly (Eidhof 2018).
- p. 17, line 24: "ARH"
- p. 17, line 27: "hydrolytic activity towards N-glycosidic bonds"
- p. 17, line 30: "hepatocellular carcinoma"
- p. 19, line 2: "truncations/mutations predictably affect protein stability"
- p. 19, line 25: "The exotoxins group encompasses a variety ..."
- p. 19, line 33: "... the latter encompassing an additional two subgroups ..."
- p. 20, line 11: "Pertussis toxin acts towards virtually all mammalian cell types ..."
- p. 20, line 14: ".. pathogenic mechanisms as well as the proteins substrate(s) of this toxin remain unknown."
- p. 20, line 17: "non-polymerised form of actin"
- p. 20, line 28: "In addition, a very intriguing class of bARTTs ..."
- p. 21, line 3: I suggest splitting this sentence, starting the second sentence with "Among them, the only known module to exploit the ADPr system ..."
- p. 21, line 5: "DarT is an ART able ..." (lose "the" in the beginning of the sentence)
- p. 21, line 7: "hydrolyses"

Decision letter (RSOB-19-0041.R0)

12-Mar-2019

Dear Dr Ahel,

We are pleased to inform you that your manuscript RSOB-19-0041 entitled "ADP-ribosylation signalling and human disease" has been accepted by the Editor for publication in Open Biology. The reviewer(s) have recommended publication, but also suggest some minor revisions to your manuscript. Therefore, we invite you to respond to the reviewer(s)' comments and revise your manuscript.

Please submit the revised version of your manuscript within 7 days. If you do not think you will be able to meet this date please let us know immediately and we can extend this deadline for you.

To revise your manuscript, log into https://mc.manuscriptcentral.com/rsob and enter your Author Centre, where you will find your manuscript title listed under "Manuscripts with Decisions." Under "Actions," click on "Create a Revision." Your manuscript number has been appended to denote a revision.

You will be unable to make your revisions on the originally submitted version of the manuscript. Instead, please revise your manuscript and upload a new version through your Author Centre.

When submitting your revised manuscript, you will be able to respond to the comments made by the referee(s) and upload a file "Response to Referees" in "Section 6 - File Upload". You can use this to document any changes you make to the original manuscript. In order to expedite the processing of the revised manuscript, please be as specific as possible in your response to the referee(s).
Please see our detailed instructions for revision requirements
https://royalsociety.org/journals/authors/author-guidelines/.

Before uploading your revised files please make sure that you have:

1) A text file of the manuscript (doc, txt, rtf or tex), including the references, tables (including captions) and figure captions. Please remove any tracked changes from the text before submission. PDF files are not an accepted format for the "Main Document".

2) A separate electronic file of each figure (tiff, EPS or print-quality PDF preferred). The format should be produced directly from original creation package, or original software format. Please note that PowerPoint files are not accepted.

3) Electronic supplementary material: this should be contained in a separate file from the main text and meet our ESM criteria (see http://royalsocietypublishing.org/instructions-authors#question5). All supplementary materials accompanying an accepted article will be treated as in their final form. They will be published alongside the paper on the journal website and posted on the online figshare repository. Files on figshare will be made available approximately one week before the accompanying article so that the supplementary material can be attributed a unique DOI.

Online supplementary material will also carry the title and description provided during submission, so please ensure these are accurate and informative. Note that the Royal Society will not edit or typeset supplementary material and it will be hosted as provided. Please ensure that

the supplementary material includes the paper details (authors, title, journal name, article DOI). Your article DOI will be 10.1098/rsob.2016[last 4 digits of e.g. 10.1098/rsob.20160049].

4) A media summary: a short non-technical summary (up to 100 words) of the key findings/importance of your manuscript. Please try to write in simple English, avoid jargon, explain the importance of the topic, outline the main implications and describe why this topic is newsworthy.

Images
We require suitable relevant images to appear alongside published articles. Do you have an image we could use? Images should have a resolution of at least 300 dpi, if possible.

Data-Sharing
It is a condition of publication that data supporting your paper are made available. Data should be made available either in the electronic supplementary material or through an appropriate repository. Details of how to access data should be included in your paper. Please see http://royalsocietypublishing.org/site/authors/policy.xhtml#question6 for more details.

Data accessibility section
To ensure archived data are available to readers, authors should include a 'data accessibility' section immediately after the acknowledgements section. This should list the database and accession number for all data from the article that has been made publicly available, for instance:
• DNA sequences: Genbank accessions F234391-F234402
• Phylogenetic data: TreeBASE accession number S9123
• Final DNA sequence assembly uploaded as online supplemental material
• Climate data and MaxEnt input files: Dryad doi:10.5521/dryad.12311

Once again, thank you for submitting your manuscript to Open Biology, we look forward to receiving your revision. If you have any questions at all, please do not hesitate to get in touch.

Sincerely,

The Open Biology Team
mailto:openbiology@royalsociety.org

Reviewer(s)' Comments to Author:

Referee: 1

Comments to the Author(s)
The review by Palazzo et al. details the current knowledge on the disease connections of ADP-ribosylation signaling. The review is well written, easy to understand and provides a valuable source of the scientific reports that establish the role of ADP-ribosylation in various human diseases.
The authors do not specifically spell out but the review focuses on the enzymes involved in ADP-ribosylation metabolism. There is probably only one exception: GDAP2, which has not been associated with either generating or degrading ADP-ribose. The 7.4 GDAP2 section is quite short and I wonder if other ADP-ribose readers associated with disease could be mentioned there, such as the macro histones and Alc1.

Regarding disease and in particular the hope of alleviating it, the therapeutic potential through targeting the players of ADP-ribosylation signaling could be discussed. Especially the review of the current status of developing selective small-molecules and the potential challenges based on the known structures and available inhibitors of the enzymes to be targeted would be a nice addition.
A table detailing the proteins and the associated diseases with references would be a great help for the reader looking for specific questions.
In section 7.6, it is written that the fold and catalytic mechanism of ARH3 are completely different from PARG. While there are several original publications to support such statement, authors could briefly mention some information they find important, such as how that translates to similarities and differences in function or the potential for their therapeutic targeting.

Minor comments on typos:
P2L19 "transferred onto on nucleic" omit "on"
P5L24 "The member 1 of the mammalian PARPs" does not read well, please rephrase.
P7L32 " characterized by; aggregation" omit semi-colon.
P8L16 "imbalance" might be better than "unbalance".
P8L32 "splice variants" instead of "splicing variants"
P9L25 Inflammatory bowel disease is a type of colitis, I suggest omitting "colitis".
P9L26 Not all mentioned are causes but also manifestations. Infection, ischemia and allergic reactions are causes the others not.
P10L4 "Alkylating" instead of "alkylation"
P10L33 Could authors rephrase "passing though"?
P12L9 and L12 Delete "s" from "plays" and "proteins"
P12L27 Delete the surplus "the"
P14L21 kilobase
P21L7 "hydrolyzes" instead of "hydrolases"

Referee: 2

Comments to the Author(s)
This excellent review will no doubt be well-received by anyone interested in a comprehensive yet concise summary of disease-relevant functions of ADP-ribosylation. The manuscript refers to numerous recent studies, covers a wide range of enzymes in different systems, including bacterial toxins/antitoxins and touches upon the function of ADP-ribosylation in viral infection. I enjoyed reading this unique manuscript and would be happy to see it published in Open Biology.

I have a number of suggestions for revising the manuscript.

Major points:
- The inhibition of DNA-dependent PARP enzymes (most notably PARP1) in BRCA-deficient cancers is not discussed. This is certainly one of the most tangible successes in targeting PARPs in the clinic. Mechanistic insights are being gained: trapped PARP1 on DNA breaks appear to be the toxic lesion initiating synthetic lethality. I think it is rather important to discuss this. If the authors would like to limit the discussion, they can refer the reader to other excellent reviews on the topic.
- The field suffers from a somewhat clumsy nomenclature, even referring to mono-ADP-ribosyltransferases as PARPs. (See also Figure 1: the term "mono-PARP" is contradictory in itself.) The review would be an opportunity to use a nomenclature that is clearer to the outsider. Many authors prefer the old, inconsistent nomenclature for historic reasons, but this may impair clarity for readers not yet familiar with the PARP field. This will be the authors' call.

- p.9, paragraph 1: There is an apparent contradition, or at least a potential source for misunderstanding. In line 7, the authors state that "inhibition of Tankyrases does not alter the overall formation of SGs". In contrast, in lines 10 and 11, the authors point out that PARP inhibition "reduces the accumulation of TDP-43 ...". Are SGs still being observed?
- Two major incentives for targeting the tankyrase proteins by small molecule inhibitors are to control their function in canonical Wnt signalling (for example related to colorectal cancer) and glucose homeostasis (related to diabetes). Can the authors either briefly discuss these aspects or refer the reader to other reviews? Tankyrase has also gained attention through its roles in telomere length maintenance. A few more words on this aspect, which is also relevant to disease mechanisms, would be welcomed.
- I think the following study deserves to be discussed in the context of tankyrase, in particular since neurobiology already features prominently in the review: Fancy et al. (2011) Axin2 as regulatory and therapeutic target in newborn brain injury and remyelination. Nat. Neurosci. 14, 1009–1016.
- p. 12, paragraph 3: Tankyrase appears to impinge on a wide range of functions and have many interaction partners and substrates. The sheer number of binders/substrates, illustrated by references 160 and 150, should be referred to. Also, another study worth adding in this context is the following: Li et al. (2017) Proteomic Analysis of the Human Tankyrase Protein Interaction Network Reveals Its Role in Pexophagy. Cell Reports 20, 737–749.
- p. 12, line 8: Functions of tankyrase in HR-mediated DNA damage repair and Notch signalling are fairly recent discoveries. Therefore, it may be a bit premature to talk about "crucial roles". This may well be the case, but without a deeper mechanistic understanding, I recommend caution in the wording. Among the best understood functions of tankyrase are canonical Wnt signalling, telomere length maintenance and roles in mitosis.
- p. 12, line 13: Many tankryase-binding motifs in fact are octapeptides, as informed by peptide permutation studies (see reference 160). The consensus has also been determined in greater detail than RxxPDG (also see reference 160). Also, "non-canonical" motifs with an N-terminally displaced arginine residue are emerging (see DaRosa et al., Protein Sci, 2018 vol 27). Having said that, the particular motif in 3BP2 is indeed an example for a hexapeptide as the residue at position 8 makes minimal contributions to binding.
- p. 12, line 20: Whether PTEN is a direct tankyrase binder or substrate is still not clear.
- p. 12, paragraph 3: Although the PAR-dependent ubiquitination pathway is ubiquitous, many tankyrase binders do not become PARylated or destabilised (e.g., MCL1, GMD). The mechanistic basis for this observation is not known. The sentence in lines 21 and 22 should therefore be revised accordingly.

Minor points / stylistic suggestions / typos:
- The abbreviation ADPr is used for both ADP-ribosylation and ADP-ribose in the article. A disambiguation would be desirable.
- Abstract (p. 1): "The control of the ADPr network is vital, and dysregulation ..."
- Abstract (p. 1): "... acquired human diseases, such as several neuological disorders ..."
- p. 2, line 7: This is a conceptual point: cellular mechanisms do not evolve for a purpose; evolution is not purpose-directed.
- p. 2, line 8: "dynamically regulate protein function through chemical modification." - You do not need the word "structure" here.
- p. 2, line 32 and elsewhere (p.9, line 20): talking of "cure" is very strong. This could, for example, be exchanged by "available therapy".
- p. 3, line 27: "paragraph thirteen" - indicating the section would suffice as readers may not want to count paragraphs.
- p. 3, line 32: "extracellular"
- p. 6, line 9: "Ser-ADPr targets include ..."
- p. 6, line 10: "has been found linked to factors mediating RNA processing, chromatin modification, splicing, ..."

- p.6, line 26: "... which involves the production ..."
- p. 6, line 29: "Similar observatiuons were made ..."
- p. 6, line 30 & 31: "Moreover, PARP1 activation was shown in [...] patients who died of stroke, ..."
- p. 7, line 7: I suggest saying "We next describe the role of PARP1 activation ..."
- p. 7, line 32: "... which is characterized by aggregation ..."
- p.8, line 34: The sentence commencing with "In turn, nuclear PAR aids the translocation ..." is unclear. Can the mechanism be described somewhat more extensively?
- p. 10, line 14: "... are encoded in the human genome ..."
- p. 10, line 25: The sentence "PARP9 is recruited at the DNA damage foci ..." is unclear.
- p. 10, line 32: Likewise, the sentence "It should be noted that the oncogenic potential ..." can be written in a clearer way.
- p. 11, line 13: "... which also is a substrate of PARP14 ..."
- p. 11, line 28: "When secreted into the extracellular environment [...] and, in addition, facilitates angiogenesis ..."
- p. 12, line 28: "tankyrase substrates"
- p. 13, line 17: "OADPr" hasn't been defined.
- p. 14, line 17: "among them the MacroD2 gene"
- p. 14, line 21: "250 kilobase"
- p. 14, line 24: "It is worth mentioning ..."
- p. 14, line 25: I suggest separating the sentence and have the second sentence start with "Thus, more observations are needed to explore ..." (rather than "establish")
- p. 14, line 33: "Importantly, some MacroD2 somatic mutations ..."
- p. 15, line 7: "... phophate- [or phospho-]linked ADPr [...] [44, 47, 188, 189]"
- p. 15, line 24: The logic of the sentence "Nonetheless, the accumulation of peptides ..." is unclear.
- p. 16, line 2: "Differently from TARG1, MacroD1 and MacroD2, ..."
- p. 16, line 13: "... have not been directly linked ..."
- p. 16, line 20: "... die at the embryonic stage; however, when grown at a permissive temperature, survival is increased."
- p. 16, line 27: "PARG functions have also been linked ..."
- p. 17, line 10: "PARG has already been proposed ..."
- p. 17, line 10: I suggest splitting the sentence, starting the second sentence with "This would be particularly appropriate ..."
- p. 17, line 19: one reference is formatted incorrectly (Eidhof 2018).
- p. 17, line 24: "ARH"
- p. 17, line 27: "hydrolytic activity towards N-glycosidic bonds"
- p. 17, line 30: "hepatocellular carcinoma"
- p. 19, line 2: "truncations/mutations predictably affect protein stability"
- p. 19, line 25: "The exotoxins group encompasses a variety ..."
- p. 19, line 33: "... the latter encompassing an additional two subgroups ..."
- p. 20, line 11: "Pertussis toxin acts towards virtually all mammalian cell types ..."
- p. 20, line 14: ".. pathogenic mechanisms as well as the proteins substrate(s) of this toxin remain unknown."
- p. 20, line 17: "non-polymerised form of actin"
- p. 20, line 28: "In addition, a very intriguing class of bARTTs ..."
- p. 21, line 3: I suggest splitting this sentence, starting the second sentence with "Among them, the only known module to exploit the ADPr system ..."
- p. 21, line 5: "DarT is an ART able ..." (lose "the" in the beginning of the sentence)
- p. 21, line 7: "hydrolyses"

Decision letter (RSOB-19-0041.R1)

22-Mar-2019

Dear Dr Ahel

We are pleased to inform you that your manuscript entitled "ADP-ribosylation signalling and human disease" has been accepted by the Editor for publication in Open Biology.

You can expect to receive a proof of your article from our Production office in due course, please check your spam filter if you do not receive it within the next 10 working days. Please let us know if you are likely to be away from e-mail contact during this time.

Thank you for your fine contribution. On behalf of the Editors of Open Biology, we look forward to your continued contributions to the journal.

Sincerely,

The Open Biology Team
mailto: openbiology@royalsociety.org
